# Targeted tissue delivery of RNA therapeutics using antibody–oligonucleotide conjugates (AOCs)

**DOI:** 10.1093/nar/gkad415

**Published:** 2023-05-24

**Authors:** Barbora Malecova, Rob S Burke, Michael Cochran, Michael D Hood, Rachel Johns, Philip R Kovach, Venkata R Doppalapudi, Gulin Erdogan, J Danny Arias, Beatrice Darimont, Christopher D Miller, Hanhua Huang, Andrew Geall, Husam S Younis, Arthur A Levin

**Affiliations:** Avidity Biosciences, Inc., 10578 Science Center Drive Suite 125, San Diego, CA 92121, USA; Seawolf Therapeutics, One Sansome Street Suite 3630, San Francisco, CA 94104, USA; Avidity Biosciences, Inc., 10578 Science Center Drive Suite 125, San Diego, CA 92121, USA; Avidity Biosciences, Inc., 10578 Science Center Drive Suite 125, San Diego, CA 92121, USA; Avidity Biosciences, Inc., 10578 Science Center Drive Suite 125, San Diego, CA 92121, USA; Avidity Biosciences, Inc., 10578 Science Center Drive Suite 125, San Diego, CA 92121, USA; Avidity Biosciences, Inc., 10578 Science Center Drive Suite 125, San Diego, CA 92121, USA; Avidity Biosciences, Inc., 10578 Science Center Drive Suite 125, San Diego, CA 92121, USA; Avidity Biosciences, Inc., 10578 Science Center Drive Suite 125, San Diego, CA 92121, USA; CYTOO, 7 Parv. Louis Néel CS 20050, 38040 Grenoble, France; California Northstate University College of Medicine, 9700 W Taron Dr, Elk Grove, CA 95757, USA; Avidity Biosciences, Inc., 10578 Science Center Drive Suite 125, San Diego, CA 92121, USA; Replicate Biosciences, 10210 Campus Point Dr, Suite 150, San Diego, CA 92121, USA; Avidity Biosciences, Inc., 10578 Science Center Drive Suite 125, San Diego, CA 92121, USA; Avidity Biosciences, Inc., 10578 Science Center Drive Suite 125, San Diego, CA 92121, USA

## Abstract

Although targeting TfR1 to deliver oligonucleotides to skeletal muscle has been demonstrated in rodents, effectiveness and pharmacokinetic/pharmacodynamic (PKPD) properties remained unknown in higher species. We developed antibody–oligonucleotide conjugates (AOCs) towards mice or monkeys utilizing anti-TfR1 monoclonal antibodies (αTfR1) conjugated to various classes of oligonucleotides (siRNA, ASOs and PMOs). αTfR1 AOCs delivered oligonucleotides to muscle tissue in both species. In mice, αTfR1 AOCs achieved a > 15-fold higher concentration to muscle tissue than unconjugated siRNA. A single dose of an αTfR1 conjugated to an siRNA against *Ssb* mRNA produced > 75% *Ssb* mRNA reduction in mice and monkeys, and mRNA silencing was greatest in skeletal and cardiac (striated) muscle with minimal to no activity in other major organs. In mice the EC_50_ for *Ssb* mRNA reduction in skeletal muscle was >75-fold less than in systemic tissues. Oligonucleotides conjugated to control antibodies or cholesterol produced no mRNA reduction or were 10-fold less potent, respectively. Tissue PKPD of AOCs demonstrated mRNA silencing activity primarily driven by receptor-mediated delivery in striated muscle for siRNA oligonucleotides. In mice, we show that AOC-mediated delivery is operable across various oligonucleotide modalities. AOC PKPD properties translated to higher species, providing promise for a new class of oligonucleotide therapeutics.

## Graphical Abstract

**Graphical Abstract:**
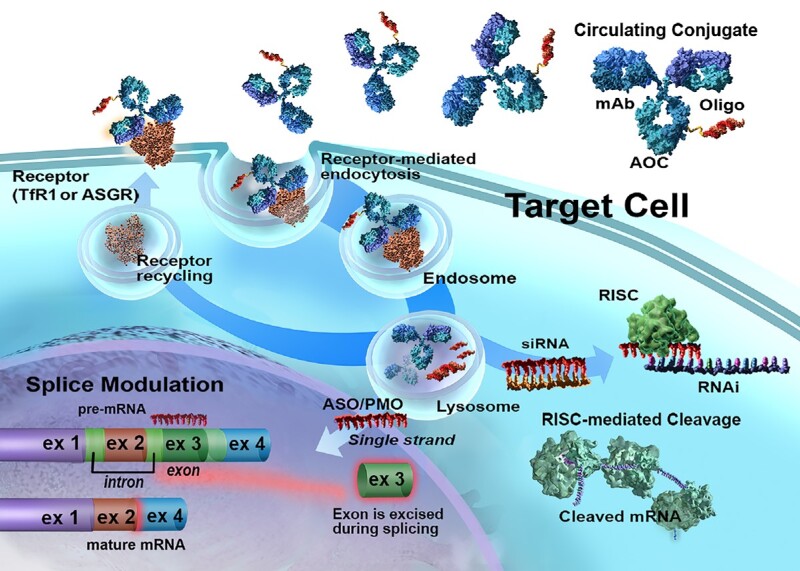

## INTRODUCTION

Oligonucleotide therapeutics are an emerging class of medicines that have transformed the way diseases are treated by targeting the underlying molecular basis of the disease. Oligonucleotides are primarily designed to bind to the target RNA to elicit multiple pharmacological mechanisms that include mRNA degradation (antisense, RNA interference, nonsense-mediated decay), skipping to promote exon inclusion or exclusion, and pre-mRNA splicing, among others (1,2). Several oligonucleotide therapies, including siRNA, ASO and PMO have been approved for treatment of cardiovascular, neuromuscular, and central nervous system diseases (2–4).

Systemic delivery of oligonucleotide-based drugs has focused on targeting the liver given the propensity for hepatocytes to productively uptake oligonucleotides for pharmacological activity (2,5). This is partly due to the relatively higher rate of blood perfusion, and discontinuous sinusoidal endothelial architecture of the liver, affording oligonucleotides to be more amenable for delivery and uptake. Although unmodified oligonucleotides have poor drug-like properties, decades of research investigating the structure–activity relationships for this broad class of drugs have identified chemical modifications that improve their pharmacokinetic and pharmacodynamic properties. Despite advances in improved stability and efficient delivery to the liver, uptake and pharmacological activity in extrahepatic tissues have limited the broad application of oligonucleotides as therapeutics (6).

Strategies that have been employed to productively deliver oligonucleotide-based drugs include complex nanoparticle formulations and conjugation to lipids, peptides, or ligands. The development of lipid nanoparticle (LNP) formulations for delivery of siRNA and mRNA enabled clinical testing of these nucleic acid-based classes of therapeutics (7). LNP formulations were successful in delivering oligonucleotides to the liver; however, delivery to other organ systems was negligible, and their safety profile was not conducive for broad applicability as treatment options for chronic diseases (8). Although significant advances have been made to LNP formulations, alternative approaches have proven superior for delivery to the liver, namely conjugation of asialoglycoprotein receptor (ASGR) ligands to oligonucleotides. *N*-Acetylgalactosamine (GalNAc) is a high-affinity ligand for ASGR that is predominately expressed on hepatic parenchymal cells. A trimer of GalNAc linked to an oligonucleotide has demonstrated up to a 10-fold improvement in potency for liver-targeting oligonucleotide therapeutics in preclinical and clinical testing (9). Although this approach does not afford extrahepatic delivery, it demonstrated that specific targeting of cell surface receptors that undergo internalization is a successful strategy for oligonucleotide delivery.

Monoclonal antibodies (mAbs) are well-established single-agent therapeutics, given their specificity and sensitivity for extracellular targets. mAbs have also been utilized for the targeted delivery of small-molecule drugs (antibody–drug conjugates, [ADC]), particularly in oncology. ADCs provide specific targeting of a chemotherapeutic to tumor cells while reducing toxicity to normal cells and tissues (10). The successful application of ADCs in oncology further supports receptor-mediated internalization as a means for effective drug delivery. In this regard, the ability of mAbs to enable specific targeting of oligonucleotides to tissues and cells to expand the therapeutic potential of this class of drugs beyond the liver has been described (11). Such molecules consist of an antibody and an oligonucleotide conjugated through a linker. The conjugation of oligonucleotides to mAbs offers a flexible and tailored approach where the antibody targets a cell type of interest to deliver an oligonucleotide for the desired disease-specific gene target. Indeed, Cuellar *et al.* demonstrated the feasibility of conjugating siRNA oligonucleotides to mAbs and producing conjugates that are active and stable *in vitro* for several mAb receptor and siRNA gene targets. In brief, the mAb-siRNA conjugates maintained comparable binding affinity and gene silencing relative to the mAb or siRNA alone *in vitro* (12). However, the *in vivo* activity of the mAb-siRNA constructs in tumor tissue was modest (12). Nonetheless, this and other early research provided evidence for the antibody-siRNA constructs to target and enter cell types outside of the liver, including skeletal muscle (13). Sugo *et al.* described the utility of targeting the transferrin receptor 1 (TfR1) to deliver oligonucleotides to skeletal muscle and heart tissue. They demonstrated in rodents a reduction of *Mstn* mRNA expression in muscle with an αTfR1-antibody fragment (Fab)-linker-siRNA conjugate. Given this progress, we investigated the translation of these observations to higher species in non-human primates using monoclonal antibody–oligonucleotide conjugates (AOC). We engineered AOCs targeting mouse ASGR (αASGR) and mouse or human TfR1 (αTfR1) receptors to facilitate the functional delivery of oligonucleotides to liver and muscle cells, respectively, across various mRNA targets with siRNA, ASO or PMO oligonucleotide modalities to further investigate the therapeutic potential and PKPD properties of the AOC platform.

We demonstrate that αTfR1-targeting AOCs are pharmacologically active in non-human primates. Furthermore, the PKPD properties of the AOC platform demonstrate the dependence of receptor-mediated delivery for activity in skeletal and cardiac muscle with apparent selective activity in striated muscle as compared to other tissues. This potential broad approach to enable productive oligonucleotide delivery to tissues beyond the liver provides promise for a new class of oligonucleotide therapeutics for the treatment of muscle diseases, among others, with high unmet need.

## MATERIALS AND METHODS

### Oligonucleotide synthesis

The oligonucleotide (siRNA and ASO) sequences were assembled on solid phase using well-described and standard phosphoramidite methodology and purified by high-performance liquid chromatography. The sugar modifications (2′-fluoro, 2′-*O*-methyl and LNA), and backbone modifications (phosphorothioates) that are well described in the fields of RNA interference (RNAi) and antisense were used to optimize the potency of the siRNA and ASO classes of oligonucleotides (14,15). The siRNA passenger strand and the ASO used for conjugation were synthesized with a C6 amino linker at the 5′-end. A trivalent GalNAc (purchased from AxoLabs, Kulmback, Germany) was conjugated to the siRNA at the 5′-end of the passenger strand. PMOs were purchased from Gene Tools LLC (Philomath, OR).

### Antibody generation

A rat IgG2a anti-mouse TfR1 antibody (αTfR1; Bio X Cell, Lebannon, NH) or a rabbit IgG anti-mouse ASGR1 antibody (αASGR; SinoBiological, Wayne, PA) was utilized for AOC synthesis in rodent experiments. A mouse IgG2 anti-human TfR1 that is cross-reactive in cynomolgus monkeys was developed (αhTfR1, Supplementary Table S1) for experiments in monkeys. A rabbit isotype control antibody (αIgG) was utilized as a non-receptor-targeting control AOC (CrownBio, Santa Clara, CA). For the TfR1 and ASGR antibodies, the binding affinity to the respective receptor target was 42 and 4.8 pM, respectively. Importantly, conjugation of an oligonucleotide had no impact on antibody binding to the target receptor (Supplementary Figure S1).

### AOC synthesis

AOCs were generated using a standard random cysteine conjugation method (16). The interchain disulfide bonds of the antibody were partially reduced with tris(2-carboxyethyl)phosphine (TCEP) prior to conjugation with a maleimide linker-oligonucleotide. The ASO and siRNA conjugate reaction mixtures were purified using strong anion exchange chromatography to ensure an average drug-to-antibody ratio (DAR) of 1 for siRNA or 2.5 for ASO AOCs. PMO AOCs were purified by hydrophobic interaction chromatography with an average DAR of 1.5. The composition of all test articles is listed in Supplementary Table S2.

### Animal treatment

All animal studies were conducted following protocols approved by the Institutional Animal Care and Use Committee (IACUC), in accordance with the regulations outlined in the USDA Animal Welfare Act as well as the ‘Guide for the Care and Use of Laboratory Animals’ (17).

Male or female CD-1 mice (Envigo, Indianapolis, IN), approximately 6–8 weeks old, were allowed to acclimate for at least 1 week prior to dosing. AOCs were administered by intravenous (IV) bolus injection to the tail vein (5 mL/kg) at the described dose. At the specified timepoints post a single dose, blood was collected and processed to plasma for assessment of AOC drug concentrations. At designated timepoints, animals were euthanized with carbon dioxide followed by cervical dislocation for collection of tissues (skeletal muscles, heart, liver, kidney, among others) that were frozen in dry ice for assessment of oligonucleotide tissue concentrations or pharmacodynamic endpoints.

Naïve male cynomolgus monkeys (approximately 2–4 years old, 2–3 kg body weight, Cambodia-sourced) were administered a single IV infusion (30 min at 5 ml/kg) at 6 mg/kg. Animals were euthanized using Euthasol (Virbac AH Inc., Fort Worth, TX) on Day 28 post dose and skeletal muscle as well as systemic tissues (25–40 mg) were collected. Samples were frozen in liquid nitrogen for evaluation of pharmacodynamic endpoints and oligonucleotide tissue concentrations. The in-life portion of the cynomolgus monkey experiments were conducted at Altasciences (Everett, WA).

The dose is expressed as the oligonucleotide component for all test molecules evaluated for all *in vivo* studies described, to allow for appropriate comparison across studies and classes of oligonucleotide chemistries.

### Pharmacodynamic evaluation in tissue

Tissue was processed for RNA extraction using the Zymo Direct-zol-96 RNA purification kit (Zymo Research Corporation, Irvine, CA). Once RNA quality was confirmed, cDNA was generated from purified RNA and used in quantitative reverse transcription PCR (RT-qPCR) analysis for gene expression for each of the targets of interests (18). The reference mRNA transcripts for *Ppib* or *AHSA1* were used in ΔΔCt calculations for mouse and cynomolgus monkey tissue samples, respectively (19). For exon skipping, nested RT-PCR was utilized as previously described (20). Briefly, primers in exon 20 (5′-ACCCAGTCTACCACCCTATC) and exon 25 (5′-CTCTTTATCTTCTGCCCACCTT) were used to amplify both skipped and unskipped mRNA transcripts. PCR products were analyzed in 4% TAE agarose gel electrophoresis to resolve skipped and unskipped transcripts. Bands were quantified using image densitometry.

To evaluate siRNA concentrations in plasma or tissues, custom stem–loop-RT-qPCR assays were developed as described previously (21). For each siRNA, a specific stem–loop-RT-qPCR assay was designed to quantify the guide strand, using custom DNA forward, reverse, and reverse transcription primers (Integrated DNA Technologies, Coralville, IA) and a custom Taqman probe (ThermoFisher Scientific, Waltham, MA). To evaluate ASO concentrations in tissues, a custom oligonucleotide probe hybridization Meso Scale Discovery (MSD) electrochemiluminescent (ECL) assay was developed. The probe was complementary to the ASO sequence and contained flanking locked nucleic acid residues with internal DNA residues. The assay followed a similar format to previously described methods (22) but was converted to MSD format with a ruthenium-labeled detector.

### Statistical analysis

GraphPad Prism software was utilized for all descriptive and statistical analyses. As appropriate, one- or two-way ANOVA or paired t-tests were performed for the datasets analyzed. In the case of ANOVA, an appropriate *post hoc* test was utilized to determine the differences among the treatment groups. Significant differences were defined as *P* < 0.05.

## RESULTS

### AOC-mediated tissue delivery

**Figure 1.**
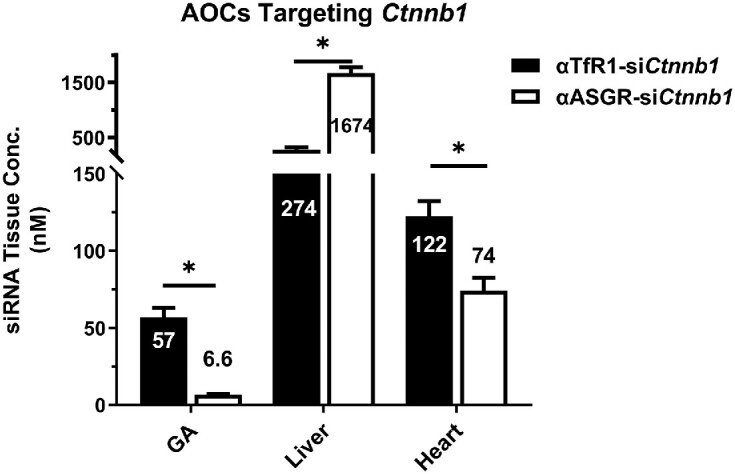
AOC-mediated siRNA tissue delivery is antibody-dependent. Female CD-1 mice were treated with a single IV dose of antibody-siRNA conjugates; each composed of an antibody targeting either mouse ASGR or mouse TfR1 and an siRNA targeting *Ctnnb1* mRNA at 3 mg/kg. GA, heart, and liver samples were collected 4 days post dose, and siRNA concentration was determined using stem-loop qPCR (normalized to tissue weight, mean ± SEM; *N* = 4). Statistical analysis was performed using two-way ANOVA with Bonferroni *post hoc* test. *Indicates statistical difference at *P*< 0.05.

The selective delivery of oligonucleotide therapeutics to target tissues of interest was evaluated for the AOC platform in mice. For these experiments, an siRNA-targeting catenin beta 1 (*Ctnnb1*) mRNA (si*Ctnnb1*) was conjugated to an antibody targeting mouse ASGR (αASGR-si*Ctnnb1*) or mouse TfR1 (αTfR1-si*Ctnnb1*) for liver or skeletal muscle siRNA delivery, respectively. The tissue concentrations of si*Ctnnb1* in gastrocnemius (GA) muscle were ∼9-fold greater with the αTfR1-si*Ctnnb1* as compared with the αASGR-si*Ctnnb1*; in contrast, ∼6-fold greater liver concentrations were delivered with the αASGR-si*Ctnnb1* (Figure 1). These results are consistent with expression of TfR1 in muscle and ASGR primarily in the liver (23,24). Although TfR1 is minimally expressed in the liver, meaningful concentrations of si*Ctnnb1* are present in this tissue, which is suggestive of non-receptor-mediated uptake. Non-receptor-mediated uptake may also be evident in the heart, given that meaningful concentrations of siRNA were observed with the αASGR-si*Ctnnb1*, despite a lack of ASGR expression in the heart. An ∼1.5-fold increase in siRNA concentration in the heart with the αTfR1-si*Ctnnb1* indicates receptor-mediated delivery to the heart may also be afforded by TfR1 targeting (Figure 1). Oligonucleotides are known to bind non-specifically to proteins and cell surface receptors, and, in part, may account for some of the uptake of si*Ctnnb1* in tissues that may not be driven by the antibody binding to its respective target, particularly the liver. Nonetheless, non-receptor-mediated uptake of the antibody (e.g. neonatal Fc receptor recycling) may also be a contributory factor.

### AOC-mediated activity in the liver in the mouse

**Figure 2.**
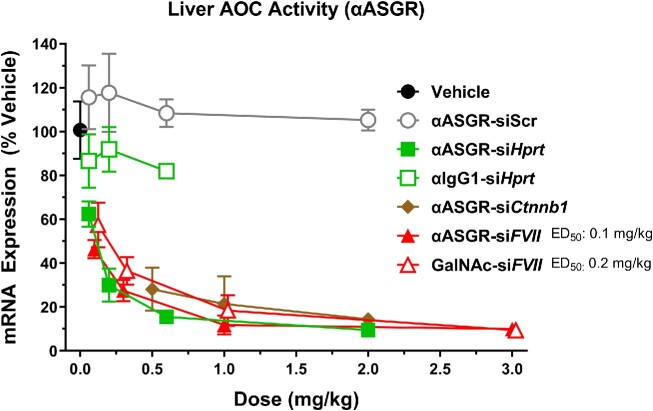
αASGR AOCs produce mRNA reduction in liver across multiple gene targets. Female CD-1 mice were treated with a single IV dose of AOCs composed of an antibody targeting mouse ASGR and an siRNA targeting either *Hprt, Ctnnb1*, or *FVII* mRNAs at indicated doses. A GalNAc conjugated to an siRNA targeting *FVII* mRNA was also evaluated following a single IV dose. Liver was collected 4 days post dose, and mRNA expression was analyzed by RT-qPCR. *Hprt, Ctnnb1*, and *FVII* mRNA expression was normalized to that of a reference gene, *Ppib*. Data are represented as percent of vehicle control (mean ± SEM; *N* = 4 for treated groups, *N* = 5 for vehicle groups). Statistical analysis was performed using one-way ANOVA with Dunnett's *post hoc* test. Statistical difference relative to vehicle control at *P*< 0.05 was observed for the αASGR-si*Hprt*, αASGR-siFVII, GalNAc-siFVII, and αASGR-siCtnnb1 at all doses evaluated.

Conjugation of the GalNAc ligand to oligonucleotides is an effective delivery mechanism of siRNAs to the liver, given the selective expression of ASGR on hepatocytes (9). We compared the siRNA-mediated potency of αASGR AOCs across various mRNA targets relative to GalNAc-mediated delivery in mice. The AOC molecules targeting three liver-expressing targets (hypoxanthine-guanine phosphoribosyltransferase [si*Hprt*], si*Ctnnb1* and factor VII [si*FVII*]) produced dose-dependent reduction in hepatic mRNA expression of each transcript with comparable activity (Figure 2). Furthermore, mRNA reduction for these molecules was receptor-dependent, indicated by a lack of activity when conjugating the si*Hprt* to an isotype control antibody immunoglobulin G1 (αIgG1), αIgG1-si*Hprt*. A scrambled siRNA sequence (siScr) with no known mRNA target also did not modulate the expression of any of the target genes evaluated. The potency for the αASGR AOCs appeared to be comparable with that of GalNAc-mediated delivery given that αASGR-si*FVII* had an ED_50_ of 0.1 mg/kg while a GalNAc conjugate of the identical siRNA had an ED_50_ of 0.2 mg/kg.

### AOC-mediated activity in muscle tissue in the mouse

**Figure 3.**
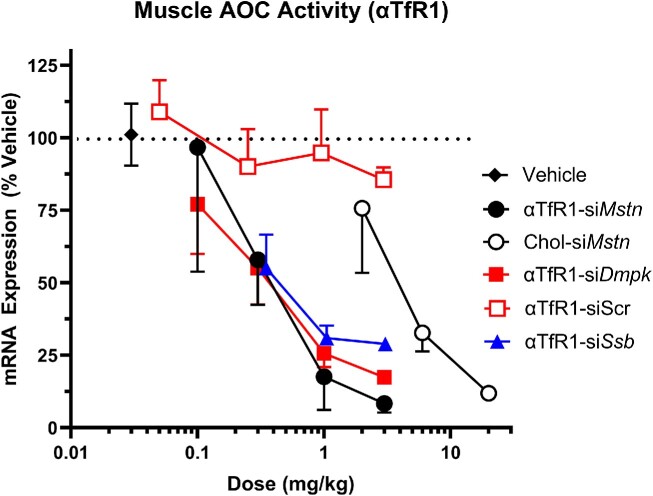
αTfR1 AOCs produce mRNA reduction in muscle across multiple gene targets. Female CD-1 mice were treated with a single IV dose of AOCs composed of an antibody targeting mouse TfR1 and an siRNA targeting either a scrambled oligonucleotide sequence (Scr), *Dmpk, Mstn*, or *Ssb* mRNAs at indicated doses. Cholesterol (Chol) conjugated to an siRNA targeting *Mstn* mRNA was also evaluated. GA muscle was isolated 4 days post dose of the αTfR1-si*Mstn* and 1 week post dose of the αTfR1-si*Dmpk* and αTfR1-si*Ssb*, and mRNA expression was analyzed by RT-qPCR. *Dmpk, Mstn*, and *Ssb* mRNA expression was normalized to that of a reference gene, *Ppib*. Data are represented as percent of vehicle control (mean ± SEM; *N* = 4 for treated groups, *N* = 5 for vehicle groups). Statistical analysis was performed using one-way ANOVA and Dunnett's *post hoc* test. Statistical difference relative to vehicle control at *P*< 0.05 was observed for all groups except αTfR1-siScr, αTfR1-si*Mstn* at 0.1 mg/kg, and Chol-si*Mstn* at 2 mg/kg.

Oligonucleotide pharmacological mechanisms of action may address a broad range of muscle diseases; however, oligonucleotide delivery to striated muscle tissue is a key limitation for this class of molecules. Thus, we developed an AOC to mediate oligonucleotide delivery to muscle tissue using αTfR1. To determine if the delivered siRNA into muscle is pharmacologically active, several αTfR1 AOCs conjugated to siRNAs targeting muscle-expressed genes myostatin (si*Mstn*), myotonic dystrophy protein kinase (si*Dmpk*), or small RNA binding exonuclease protection factor La (si*Ssb*) were tested *in vivo* in mice. All AOCs evaluated (αTfR1-si*Mstn*, αTfR1-si*Dmpk*, and αTfR1-si*Ssb*) produced dose-dependent reduction of mRNA expression for each target gene in GA muscle (Figure 3). Reduction of mRNA expression was > 80% for αTfR1-si*Mstn* and αTfR1-si*Dmpk* siRNAs, demonstrating that marked pharmacological activity could be afforded by αTfR1-mediated delivery to muscle. Moreover, αTfR1-si*Mstn* was at least 10-fold more potent than conjugation of cholesterol (Chol) ligand to the siRNA (Chol-si*Mstn*), demonstrating the higher efficiency of the AOC platform relative to conjugation of a non-targeted ligand.

**Figure 4.**
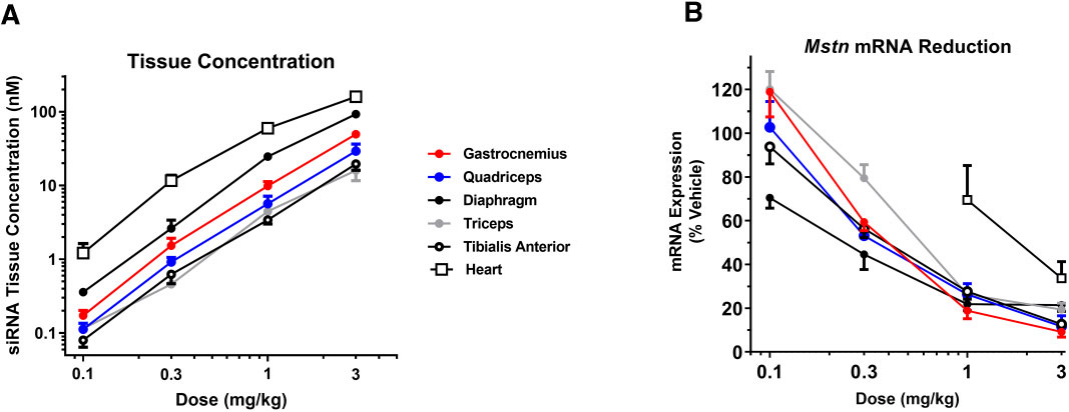
αTfR1-si*Mstn* produced dose-dependent increases in siRNA tissue concentration and *Mstn* mRNA reduction in a broad panel of skeletal muscles and heart. Female CD-1 mice were treated with a single IV dose of αTfR1-si*Mstn* at indicated doses. Muscle tissue was isolated 7 days post dose. mRNA expression was analyzed by RT-qPCR, and siRNA concentration was analyzed by stem-loop qPCR (data normalized to tissue weight). *Mstn* mRNA expression was normalized to that of a reference gene, *Ppib*. mRNA data are represented as percent of vehicle control (mean ± SEM; *N* = 4 for treated groups, *N* = 5 for vehicle group). Statistical analysis was performed using one-way ANOVA and Dunnett's *post hoc* test. Statistical difference relative to vehicle control at *P*< 0.05 was observed for all treatments except for 0.1 mg/kg dose in tibialis anterior, gastrocnemius, quadriceps, triceps, and 0.3 mg/kg in triceps.

Given the relatively broad protein expression of TfR1 (https://www.proteinatlas.org/ ENSG00000072274-TFRC/tissue), we evaluated the uptake and activity of αTfR1-si*Mstn* and αTfR1-si*Ssb* across a broad panel of skeletal muscles and systemic tissues. Indeed, αTfR1-si*Mstn* produced dose-dependent siRNA delivery in all skeletal muscles evaluated (Figure 4). siRNA concentrations ranged from 10 to 100 nM in muscle tissue at the highest dose tested (3 mg/kg). *Mstn* mRNA expression was similarly reduced in a concentration-dependent manner in muscle tissue, where activity was evident in the diaphragm as low as 0.1 mg/kg. At doses ≥1 mg/kg, near-maximal reductions in *Mstn* mRNA expression (≥80% relative to vehicle control) were produced in all skeletal muscles evaluated. To demonstrate the tissue specificity for the αTfR1 AOC, a gene target with ubiquitous expression, *Ssb* mRNA, was evaluated. The αTfR1-si*Ssb* was the most potent in muscle for *Ssb* mRNA reduction with an EC_50_ of 0.4 nM in GA muscle (Table 1). In contrast, mRNA silencing activity was ∼10-fold less potent in the heart and ∼100-fold less potent in the liver, relative to that achieved in skeletal muscle. Minimal to no activity was evident in other systemic tissues following αTfR1-si*Ssb*, including in the kidney, spleen, and gastrointestinal tract (GI [colon]).

**Table 1.** αTfR1-si*Ssb* demonstrates tissue-selective mRNA silencing activity

Tissue	EC_50_ (nM)
Skeletal muscle (GA)	0.3
Heart	4
Liver	24
Kidney	>100
Spleen	>100
GI (colon)	>100

### AOC-mediated oligonucleotide delivery and activity in muscle tissue in cynomolgus monkeys

**Figure 5.**
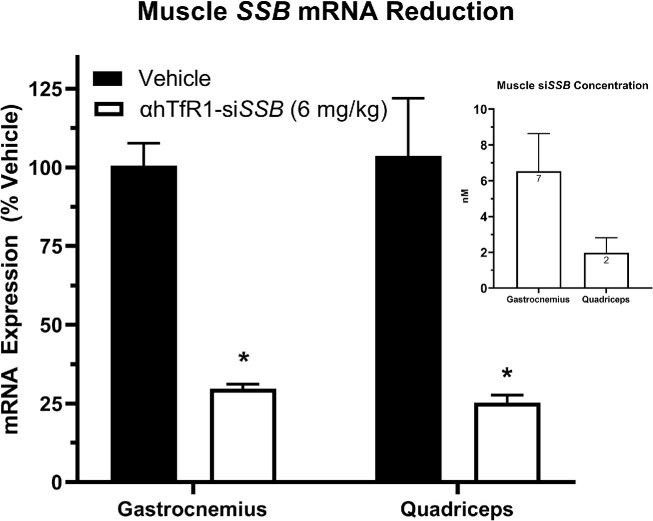
αTfR1-si*SSB* productively delivers siRNA to muscle and produces mRNA reduction in cynomolgus monkeys. Male cynomolgus monkeys of Cambodian origin were administered a single IV dose of *SSB* siRNA conjugated to an antibody targeting TfR1 at 6 mg/kg. Tissues were collected at 28 days post dose, and mRNA expression was analyzed by RT-qPCR, while siRNA tissue concentrations were determined using stem-loop RT-qPCR. *SSB* expression was normalized to reference gene activator of HSP90 ATPase activity 1 (*AHSA1*). mRNA expression (percent of vehicle control) and siRNA tissue concentration (normalized to tissue weight) data are represented as mean ± SEM (*N* = 3). Statistical analysis was performed using one-way ANOVA and Tukey's *post hoc* test. *Indicates significant difference relative to vehicle control at *P*< 0.05.

Based on the promising pharmacological activity of the AOC platform in mice, testing in non-human primates (cynomolgus monkeys) was performed to determine the translation of the pharmacokinetic and pharmacodynamic properties of this new oligonucleotide delivery platform in higher species. A human, cynomolgus monkey cross-reactive, antibody (αhTfR1) conjugated to an siRNA targeting monkey *SSB* mRNA (αhTfR1-si*SSB*) was tested at a single dose of 6 mg/kg. αhTfR1-si*SSB* productively delivered siRNA to skeletal muscle tissue, resulting in tissue concentration that ranged from 2 to 6.6 nM at Day 28 post single dose (Figure 5). The delivery of si*SSB* to muscle led to reductions of up to 75% (relative to vehicle-treated animals) in *SSB* mRNA expression in GA and quadriceps muscle (Figure 5). αhTfR1-si*SSB* demonstrated pharmacological activity in a broad panel of muscles, including the heart, at Day 28 following a single dose (Supplementary Figure S2). As observed from mouse studies, no meaningful activity was noted in liver, kidney, lung, or spleen, as *Ssb* mRNA expression was within the normal range of control in these tissues after the AOC treatment.

### AOC-mediated activity is amenable to various types of oligonucleotide chemistry

**Figure 6.**
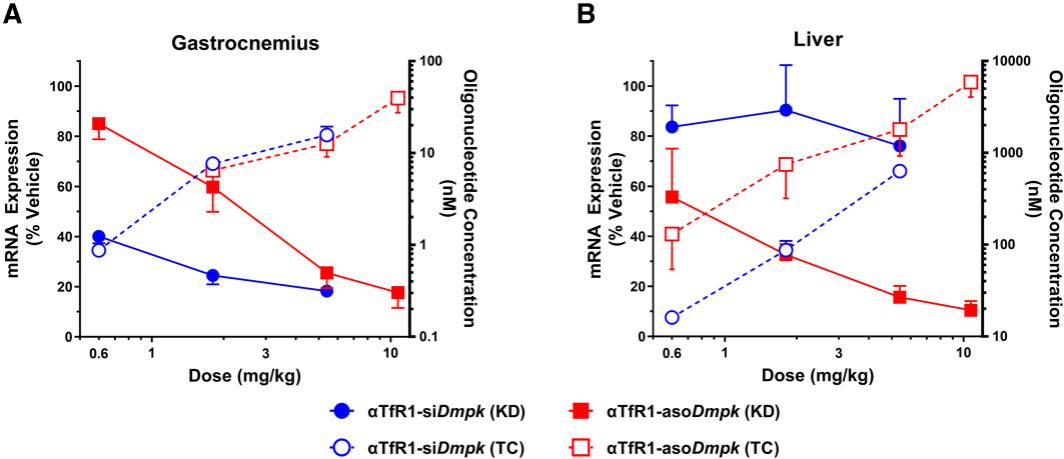
αTfR1 AOCs conjugated to siRNA or ASO oligonucleotides produce mRNA silencing activity in muscle. Male C57BL/6 mice were treated with a single IV dose of AOCs each composed of an antibody targeting mouse TfR1 conjugated to either an siRNA (αTfR1-si*Dmpk*; DAR = 1) or an ASO (αTfR1-aso*Dmpk*; DAR = average 2.5) targeting *Dmpk* mRNA at indicated doses. Tissue samples were isolated 14 days post dose, and mRNA expression was analyzed by RT-qPCR. siRNA concentration was assessed by stem-loop qPCR, and ASO concentration was assessed by oligonucleotide probe hybridization electrochemiluminescent (ECL) assay (data normalized to tissue weight). *Dmpk* mRNA expression was normalized to that of a reference gene, *Ppib*. Data are represented as percent of vehicle control (mean ± SEM; *N* = 4 for treated groups, *N* = 5 for vehicle group). Statistical analysis for KD was performed using one-way ANOVA and Dunnett's *post hoc* test. Statistical significance relative to vehicle control at *P*< 0.05 was noted for αTfR1-si*Dmpk* in muscle and for αTfR1-aso*Dmpk* in muscle and liver at all doses evaluated. To compare the αTfR1-si*Dmpk*- and αTfR1-aso*Dmpk* groups, a two-way ANOVA with Sidak's *post hoc* test was performed, and statistical significance was observed at the 0.6 and 1.8 mg/kg dose groups with *P*< 0.05. αTfR1-aso*Dmpk* (TC) in gastrocnemius at 0.6 mg/kg dose was below the limit of quantification. KD: mRNA knockdown; TC: oligonucleotide tissue concentration.

Given that oligonucleotide therapeutics comprise several chemical classes and utilize multiple mechanisms of pharmacological action, we evaluated the applicability of the AOC platform to other chemical classes, including PMO (exon splice switching) and phosphorothioate ASO (ribonuclease H1-mediated mRNA degradation) oligonucleotides. A PMO targeting exon 11 skipping of phenylalanine hydroxylase (pmo*Pah*) pre-mRNA was conjugated to αASGR to assess activity in the liver. In mice, αASGR-pmo*Pah* produced measurable skipping of exon 11 of *Pah*, with no evidence of skipping with a scrambled PMO sequence (αASGR-pmoScr) as control (Supplementary Figure S3). An ASO targeting *Dmpk* mRNA (aso*Dmpk*) was conjugated to αTfR1 to assess activity in skeletal muscle. A single dose of αTfR1-aso*Dmpk* produced dose-dependent activity with >75% mRNA reduction of *Dmpk* mRNA in GA muscle at the highest dose in mice (Figure 6A). Importantly, the unconjugated ASO (aso*Dmpk*) required a ∼25-fold greater cumulative dose to achieve comparable activity that further substantiates the efficiency of receptor-mediated delivery for pharmacological activity (Supplementary Figure S4).

We note that αTfR1-mediated delivery to muscle appeared to be independent of the oligonucleotide chemical class, given that αTfR1-si*Dmpk* and αTfR1-aso*Dmpk* achieved comparable oligonucleotide GA concentrations at similar dose levels. Surprisingly, up to 10-fold greater aso*Dmpk* was present in liver as compared with si*Dmpk*, which was associated with > 80% *Dmpk* mRNA reduction at 5.4 mg/kg (Figure 6B). αTfR1-si*Dmpk* had negligible activity in liver with no clear dose dependency at the same dose tested (5.4 mg/kg). Collectively, these data suggest that non-TfR1-mediated mechanisms may be accountable for the uptake and activity of αTfR1-aso*Dmpk* in the liver, and these properties appear to be dependent on the oligonucleotide chemical class employed.

## DISCUSSION

Advancements to improve the metabolic stability and safety profile, economize large-scale manufacturing, and identify therapeutic applications, among others, have propelled oligonucleotides as mainstream investigational therapeutics across a spectrum of diseases. A key limitation has been productive delivery to tissues, given that the liver has been the primary target tissue for systemically administered oligonucleotide therapeutics (25). Nonetheless, there has been recent progress with local administration to effectively deliver siRNAs to the central nervous system (intrathecal injection of divalent siRNA or 2′-*O*-hexadecyl [C16]–siRNA conjugates, and intravitreal injection of C16–siRNA conjugates) or lung (C16-siRNA conjugates via intranasal injection) (26,27). We report the use of monoclonal antibody-based AOCs that are administered intravenously to deliver oligonucleotides to tissues via specific receptor-mediated endocytosis, with a focus on skeletal muscle and heart. These findings, including the translation to non-human primates with αTfR1, constitute a potential breakthrough in oligonucleotide delivery.

### AOC activity is translated from rodents to non-human primates

Although prior research demonstrated the utility of TfR1 targeting for delivery of siRNAs to muscle in rodents, the translation of these findings to larger species was unknown (13). Given the successful utility of monoclonal antibodies to facilitate delivery of small-molecule cytotoxic drugs in oncology (28), we postulated that this would be a more favorable approach relative to other reported modalities (e.g. antibody fragments and peptides) (13,29). For example, the specificity and selectivity for the intended target afford biologics minimal potential for non-specific interactions relative to peptides, and neonatal Fc receptor recycling affords improved plasma pharmacokinetic properties for full-length antibodies that are generally absent for antibody fragments. In this regard, we engineered human αhTfR1 that is cynomolgus monkey cross-reactive with low pM binding affinity to TfR1 to determine the translation of utilizing TfR1 targeting to deliver oligonucleotides to muscle for pharmacological activity. Importantly, αTfR1 conjugated to the linker-oligonucleotide components did not impact binding affinity to TfR1 (Supplementary Figure S1).

We show that anti-TfR1 AOCs targeting *Ssb* mRNA in muscle are active in both mice and cynomolgus monkeys following a single dose. In the monkey, αhTfR1-si*SSB* achieved up to 70% reduction of *SSB* mRNA in multiple skeletal muscles that was comparable to that in the mouse. Importantly, αhTfR1 delivery resulted in siRNA muscle tissue concentrations 10-fold above the EC_50_, as determined in mice. In addition to skeletal muscle tissue, systemic dosing of AOCs in mice and cynomolgus monkeys resulted in sufficient delivery of oligonucleotides to the heart, leading to marked *Ssb* mRNA reduction. This is the first report demonstrating the productive delivery and pharmacological activity of an AOC in skeletal muscle and the heart in non-human primates. Although targeting *SSB* has no immediate therapeutic application, this work provides proof of concept that the AOC platform productively delivers siRNA therapeutics to muscle.

### PKPD characterization of AOCs demonstrates that receptor-mediated delivery leads to tissue-selective pharmacological activity

We show that the oligonucleotide tissue delivery mediated by antibodies to either TfR1 or ASGR is highly efficient and productive for skeletal muscle and liver, respectively. An siRNA targeting *Ctnnb1* mRNA conjugated to αTfR1 or αASGR demonstrated tissue selectivity for oligonucleotide delivery and mRNA reduction. αTfR1-si*Ctnnb1* led to a 10-fold greater uptake of si*Ctnnb1* to skeletal muscle relative to liver, while similar specificity was produced with the αASGR-si*Ctnnb1* for liver delivery. To further support the dependence on receptor-mediated activity, we demonstrated that mRNA reduction was not evident when conjugating an siRNA to a control non-specific antibody immunoglobulin G1 (αIgG1-si*Hprt*). Moreover, siRNA delivered via the AOC platform was approximately 10-fold more potent for *Mstn* mRNA reduction, relative to conjugation of oligonucleotides via a non-receptor-mediated delivery mechanism (conjugation to cholesterol). As anticipated, administration of unconjugated *Mstn* siRNA led to minimal uptake into skeletal muscle tissue, as most of the drug was rapidly cleared from systemic circulation (plasma half-life [*t*_1/2_] <0.5 hours, Supplementary Figure S5). In contrast, αTfR1-conjugated si*Mstn* (αTfR1-si*Mstn*) had much lower plasma clearance (*t*_1/2_ of ∼6 hours) with a plasma concentration–time profile comparable to the antibody alone, indicating that the AOC assumed the antibody pharmacokinetic profile. This also demonstrates the stability of the antibody–siRNA complex, given that the assays measuring total antibody or siRNA components exhibited comparable plasma exposure. The efficiency of receptor-mediated activity was also demonstrated with an αTfR1-aso*Dmpk* AOC, given that a 25-fold greater cumulative dose of the unconjugated aso*Dmpk* was required to achieve comparable *Dmpk* mRNA reduction in muscle tissue relative to a single dose of the AOC (Supplementary Figure S4).

We also noted presence of oligonucleotide in tissues that would not be anticipated based on the relatively low expression of the target receptor. For example, meaningful concentrations of si*Ctnnb1* were present in the liver and heart with the αTfR1- or αASGR-conjugated AOCs, respectively, despite relatively minimal to low expression of the target receptors in these tissues. This was more evident with the αTfR1-aso*Dmpk* AOC, with which 10-fold greater tissue concentrations of the ASO were noted in the liver compared with the siRNA AOC, αTfR1-si*Dmpk* (Figure 6B). We postulate that non-receptor-mediated uptake exists for AOCs and the extent of uptake is dependent on the conjugated oligonucleotide.

To evaluate the impact of receptor- versus non-receptor-mediated uptake on pharmacological activity, we characterized the PKPD properties of an AOC whose mRNA target expression is ubiquitous across a broad panel of tissues. In this regard, the *Ssb* mRNA target was selected, in large part, due to its relatively uniform expression across tissues, with slightly greater expression in the spleen relative to other organs evaluated (30). Indeed, in mice the EC_50_ of si*Ssb* was 0.3 nM in skeletal muscle tissue for αTfR1-si*Ssb*, while 10- to 100-fold higher concentrations were required for activity in heart and liver, respectively (Table 1). Furthermore, no meaningful reductions in the target mRNA *Ssb* were evident in other organ systems evaluated, such as the GI tract, spleen, and kidney. Our findings in mice were translated to non-human primates where no meaningful reductions in *SSB* mRNA were evident with αTfR1-si*SSB* in the non-striated muscle tissues evaluated (liver, kidney, lung and spleen). It is unlikely that the differences in mRNA silencing activity were due to differences in *SSB* expression among the tissues evaluated. Although we did not evaluate the relative mRNA or protein expression of TfR1 among various cell types within a tissue, the higher whole tissue expression of TfR1 in skeletal muscle may, in part, contribute to the tissue selectivity of pharmacological activity (12,30). Nonetheless, it does not fully explain our observations given the substantial 10-fold greater activity in skeletal muscle relative to heart at much lower siRNA concentrations. We postulate that receptor-mediated uptake leads to more productive oligonucleotide delivery and activity; however, skeletal myofibers appear to be more sensitive to activity relative to other TfR1-expressing cells. More work is needed to fully understand the potential mechanism(s) of tissue-selective pharmacological activity of AOCs, as this may include differences in endosomal processing, Ago-2-mediated mRNA cleavage, cell-specific expression of TfR1, among others, across cells/tissues. Our findings provide the foundation for further research to study and identify tissue-specific mechanisms that are critical for the activity of oligonucleotide therapeutics afforded by receptor-mediated internalization processes.

### αTfR1 AOCs support delivery and activity across multiple chemical classes of oligonucleotide therapeutics

Because oligonucleotide therapeutics include numerous mechanisms of pharmacological action across various chemical classes, we evaluated whether the AOC platform was applicable to single-stranded ASO and PMO, as was demonstrated with a double-stranded siRNA. Indeed, AOCs comprised of αTfR1 conjugated to ASO for *Dmpk* mRNA (αTfR1-aso*Dmpk*) achieved > 80% knockdown of mRNA expression in mouse skeletal muscle following a single 10 mg/kg dose. As proof of concept for modulation of pre-mRNA splicing, an antibody designed for liver delivery (αASGR) conjugated to a PMO targeting *Pah* (αASGR-pmo*Pah*) was evaluated *in vivo*. Measurable skipping of exon 11 of the *Pah* pre-mRNA was evident in the liver following a single dose of αASGR-pmo*Pah* (Supplementary Figure S3). This demonstrates the potential utility of the AOC platform to productively deliver another chemical class of oligonucleotide, PMO, *in vivo*. Given that modulation of pre-mRNA splicing by oligonucleotides has been demonstrated to be therapeutically beneficial for several disorders, especially in muscle disease, we, along with others, have reported potent and durable exon skipping activity for a PMO in an animal model of Duchenne muscular dystrophy (29,31).

Interestingly, there were notable differences in activity between the AOCs with siRNA and ASO. The siRNA was ∼3-fold more potent than the ASO in skeletal muscle, despite the concentrations of the siRNA and ASO being comparable (Figure 6). This finding suggests that the difference in potency is not likely due to differential muscle delivery of the oligonucleotide given that the same linker and αTfR1 antibody were utilized. In part, the inherent efficiency of RNA-induced silencing complex for siRNAs versus ribonuclease H1-mediated mRNA degradation by ASOs may account for the increased activity of the siRNA in skeletal muscle (32). However, in the liver, the ASO produced substantial *Dmpk* mRNA reduction, while the siRNA was devoid of meaningful activity. The potency of αTfR1-aso*Dmpk* for mRNA reduction in skeletal muscle and in the liver appears to be comparable, while the αTfR1-si*Dmpk* demonstrated apparent selectivity for productive delivery and target–mRNA silencing in muscle tissue. We did not evaluate the cellular distribution of the oligonucleotides within the liver so the target cell type(s) of the ASO is (are) uncertain. *Dmpk* mRNA expression appears higher in Kupffer cells relative to hepatocytes or endothelial cells (33), so it is reasonable to assume that Kupffer cells may be the primary cell target for the ASO within the liver. Given that ASOs have also been shown to bind to plasma membrane proteins (34), non-receptor-mediated uptake in the liver appears to be greater for this chemical class. The findings are consistent with the majority of the activity in the liver for the αTfR1-aso*Dmpk* being independent of the antibody portion of the AOC.

### Therapeutic potential of antibody-mediated oligonucleotide delivery

A key advantage of the AOC platform is that a single antibody may be utilized across multiple diseases within a tissue type where the oligonucleotide component would be interchangeable based on the therapeutic target. Thus, the safety profile of interacting and modulating TfR1 would be important to characterize as it may have implications across several potential drug candidates, especially given the broad tissue expression of TfR1 and its main function of transporting iron via transferrin into cells critical for normal hematopoiesis (35). In this regard, we engineered an αhTfR1 antibody that would not interfere with binding of transferrin to TfR1 (Supplementary Figure S6A). Nonetheless, the transient internalization of TfR1 following antibody binding may impact the potential availability of transferrin-mediated iron uptake immediately following AOC administration. However, given the relatively short plasma half-life *t*_1/2_ (<24 h), the impact of this on hematopoiesis may be transient. Importantly, we modified the antibody to lack binding to Fc gamma receptors to eliminate effector function. Indeed, we demonstrated that the αhTfR1 antibody was devoid of effector function in an *in vitro* antibody-dependent cell cytotoxicity assay (Supplementary Figure S6B).

In summary, we report a versatile approach using monoclonal AOCs for productive delivery of oligonucleotides to target muscle tissue that has been generally inaccessible to RNA therapeutics. We demonstrate the translation of the PKPD properties of the AOC platform across species from rodents to non-human primates, illustrating the consistency and potential utility of this approach in humans. In fact, the first AOC therapeutic, AOC 1001, is in early clinical testing for the treatment of myotonic dystrophy type 1 (MARINA; https://clinicaltrials.gov/ct2/show/NCT05027269). AOC 1001 is a humanized mAb targeting human TfR1 conjugated to an siRNA oligonucleotide against *DMPK* mRNA, where mutations on this gene are the cause and genetic basis of the disease. The results of clinical testing to characterize the safety and efficacy of investigational AOC therapeutics are likely to springboard the discovery and development of AOCs designed for delivery to other organs and tissue types to further extend the utility of RNA therapeutics.

## DATA AVAILABILITY

The data underlying this article are available in the article and in its online supplementary material.

## Supplementary Material

gkad415_Supplemental_File

## References

[1] Crooke S.T. , Liang X.H., Baker B.F., Crooke R.M. Antisense technology: a review. J. Biol. Chem. 2021; 296:100416. 33600796 10.1016/j.jbc.2021.100416 PMC8005817

[2] Levin A.A. Treating disease at the RNA level with oligonucleotides. N. Engl. J. Med. 2019; 380:57–70. 30601736 10.1056/NEJMra1705346

[3] Igarashi J. , Niwa Y., Sugiyama D. Research and development of oligonucleotide therapeutics in Japan for rare diseases. Future Rare Dis. 2022; 2:10.2217/frd-2021-0008.

[4] Crooke S.T. , Vickers T.A., Liang X. Phosphorothioate modified oligonucleotide–protein interactions. Nucleic Acids Res. 2020a; 48:5235–5253. 32356888 10.1093/nar/gkaa299 PMC7261153

[5] Kaczmarek J.C. , Kowalski P.S., Anderson D.G. Advances in the delivery of RNA therapeutics: from concept to clinical reality. Genome Med. 2017; 9:60. 28655327 10.1186/s13073-017-0450-0 PMC5485616

[6] Roberts T.C. , Langer R., Wood M.J.A. Advances in oligonucleotide drug delivery. Nat. Rev. Drug Discov. 2020; 19:673–694. 32782413 10.1038/s41573-020-0075-7 PMC7419031

[7] Tran P. , Weldemichael T., Liu Z., Li H.Y. Delivery of oligonucleotides: efficiency with lipid conjugation and clinical outcome. Pharmaceutics. 2022; 14:342. 35214074 10.3390/pharmaceutics14020342 PMC8879684

[8] Zatsepin T.S. , Kotelevtsev Y.V., Koteliansky V. Lipid nanoparticles for targeted siRNA delivery - going from bench to bedside. Int. J. Nanomed. 2016; 11:3077–3086. 10.2147/IJN.S106625 PMC4939975 27462152

[9] Debacker A.J. , Voutila J., Catley M., Blakey D., Habib N. Delivery of oligonucleotides to the liver with GalNAc: from research to registered therapeutic drug. Mol. Ther. 2020; 28:1759–1771. 32592692 10.1016/j.ymthe.2020.06.015 PMC7403466

[10] Khongorzul P. , Ling C.J., Khan F.U., Ihsan A.U., Zhang J. Antibody-drug conjugates: a comprehensive review. Mol. Cancer Res. 2020; 18:3–19. 31659006 10.1158/1541-7786.MCR-19-0582

[11] Benizri S. , Gissot A., Martin A., Vialet B., Grinstaff M.W., Barthélémy P. Bioconjugated oligonucleotides: recent developments and therapeutic applications. Bioconjug. Chem. 2019; 30:366–383. 30608140 10.1021/acs.bioconjchem.8b00761 PMC6766081

[12] Cuellar T.L. , Barnes D., Nelson C., Tanguay J., Yu S., Wen X., Scales S.J., Gesch J., Davis D., AvB S. et al. Systematic evaluation of antibody-mediated siRNA delivery using an industrial platform of THIOMAB–siRNA conjugates. Nucleic Acids Res. 2015; 43:1189–1203. 25550431 10.1093/nar/gku1362 PMC4333408

[13] Sugo T. , Terada M., Oikawa T., Miyata K., Nishimura S., Kenjo E., Ogasawara-Shimizu M., Makita Y., Imaichi S., Murata S. et al. Development of antibody-siRNA conjugate targeted to cardiac and skeletal muscles. J. Control Release. 2016; 237:1–13. 27369865 10.1016/j.jconrel.2016.06.036

[14] Nair J.K. , Attarwala H., Sehgal A., Wang Q., Aluri K., Zhang X., Gao M., Liu J., Indrakanti R., Schofield S. et al. Impact of enhanced metabolic stability on pharmacokinetics and pharmacodynamics of GalNAc-siRNA conjugates. Nucleic Acids Res. 2017; 45:10969–10977. 28981809 10.1093/nar/gkx818 PMC5737438

[15] Foster D.J. , Brown C.R., Shaikh S., Trapp C., Schlegel M.K., Qian K., Sehgal A., Rajeev K.G., Jadhav V., Manoharan M. et al. Advanced siRNA designs further improve *in vivo* performance of GalNAc-siRNA conjugates. Mol. Ther. 2018; 26:708–717. 29456020 10.1016/j.ymthe.2017.12.021 PMC5910670

[16] Datta-Mannan A. , Choi H., Stokell D., Tang J., Murphy A., Wroblski A., Feng Y. The properties of cysteine-conjugated antibody-drug conjugates are impacted by the IgG subclass. AAPS J. 2018; 20:103. 30255287 10.1208/s12248-018-0263-0

[17] National Research Council (US) Committee for the Update of the Guide for the Care and Use of Laboratory Animals Guide for the Care and Use of Laboratory Animals. 2011; 8th edn. Washington (DC) National Academies Press (US). 21595115

[18] Green M.R. , Sambrook J. Quantification of RNA by real-time reverse transcription-polymerase chain reaction (RT-PCR). Cold Spring Harb. Protoc. 2018; 10:847–856. 10.1101/pdb.prot095042 30275077

[19] Livak K.J. , Schmittgen T.D. Analysis of relative gene expression data using real-time quantitative PCR and the 2(-Delta Delta C(T)) method. Methods. 2001; 25:402–408. 11846609 10.1006/meth.2001.1262

[20] Jirka S.M.G. , Tanganyika-de Winter C.L., Boertje-van der Meulen J.K., van Putten M., Hiller M., Vermue R., de Visser P.C., Aartsma-Rus A. Evaluation of 2′-deoxy-2′-fluoro antisense oligonucleotides for exon skipping in Duchenne Muscular Dystrophy. Mol. Ther. Nucleic Acids. 2015; 4:e265. 10.1038/mtna.2015.39 PMC5014533 26623937

[21] Chen C. , Ridzon D.A., Broomer A.J., Zhou Z., Lee D.H., Nguyen J.T., Barbisin M., Xu N.L., Mahuvakar V.R., Andersen M.R. et al. Real-time quantification of microRNAs by stem-loop RT-PCR. Nucleic Acids Res. 2005; 33:e179. 16314309 10.1093/nar/gni178 PMC1292995

[22] Burki U. , Keane J., Blain A., O’Donovan L., Gait M.J., Laval S.H., Straub V. Development and application of an ultrasensitive hybridization-based ELISA method for the determination of peptide-conjugated phosphorodiamidate morpholino oligonucleotides. Nucleic Acid Ther. 2015; 25:275–284. 26176274 10.1089/nat.2014.0528 PMC4576940

[23] Barrientos T. , Laothamatas I., Koves T.R., Soderblom E.J., Bryan M., Moseley M.A., Muoio D.M., Andrews N.C. Metabolic catastrophe in mice lacking transferrin receptor in muscle. EBioMedicine. 2015; 2:1705–1717. 26870796 10.1016/j.ebiom.2015.09.041 PMC4740293

[24] D’Souza A.A. , Devarajan P.V. Asialoglycoprotein receptor mediated hepatocyte targeting – strategies and applications. J. Control Release. 2015; 203:126–139. 25701309 10.1016/j.jconrel.2015.02.022

[25] Moumné L. , Marie A., Crouvezier N. Oligonucleotide therapeutics: from discovery and development to patentability. Pharmaceutics. 2022; 14:260. 35213992 10.3390/pharmaceutics14020260 PMC8876811

[26] Alterman J.F. , Godinho B., Hassler M.R., Ferguson C.M., Echeverria D., Sapp E., Haraszti R.A., Coles A.H., Conroy F., Miller R. et al. A divalent siRNA chemical scaffold for potent and sustained modulation of gene expression throughout the central nervous system. Nat. Biotechnol. 2019; 37:884–894. 31375812 10.1038/s41587-019-0205-0 PMC6879195

[27] Brown K.M. , Nair J.K., Janas M.M., Anglero-Rodriguez Y.I., Dang L.T.H., Peng H., Theile C.S., Castellanos-Rizaldos E., Brown C., Foster D. et al. Expanding rnai therapeutics to extrahepatic tissues with lipophilic conjugates. Nat. Biotechnol. 2022; 40:1500–1508. 35654979 10.1038/s41587-022-01334-x

[28] Dean A.Q. , Luo S., Twomey J.D., Zhang B. Targeting cancer with antibody–drug conjugates: promises and challenges. MAbs. 2021; 13:1951427. 34291723 10.1080/19420862.2021.1951427 PMC8300931

[29] Desjardins C.A. , Yao M., Hall J., O’Donnell E., Venkatesan R., Spring S., Wen A., Hsia N., Shen P., Russo R. et al. Enhanced exon skipping and prolonged dystrophin restoration achieved by TfR1-targeted delivery of antisense oligonucleotide using FORCE conjugation in mdx mice. Nucleic Acids Res. 2022; 50:11401–11414. 35944903 10.1093/nar/gkac641 PMC9723632

[30] Li B. , Qing T., Zhu J., Wen Z., Yu Y., Fukumura R., Zheng Y., Gondo Y., Shi L. A comprehensive mouse transcriptomic BodyMap across 17 tissues by RNA-seq. Sci. Rep. 2017; 7:4200. 28646208 10.1038/s41598-017-04520-z PMC5482823

[31] Karamanlidis G. , Etxaniz U., Diaz M., Bhardwaj R., Tyaglo O., Lemoine K., Missinato M.A., Anderson A., Kovach P., Marks I. et al. Antibody-oligonucleotide conjugates (AOCs) demonstrate potent and durable exon skipping and dystrophin restoration in a mouse model of Duchenne muscular dystrophy. Neurology. 2022; 98(Suppl. 18):1761.

[32] Garber K. Worth the RISC?. Nat. Biotechnol. 2017; 35:198–202. 28244998 10.1038/nbt.3810

[33] The Tabula Muris Consortium A single cell transcriptomic atlas characterizes aging tissues in the mouse. Nature. 2020; 583:590–595. 32669714 10.1038/s41586-020-2496-1 PMC8240505

[34] Crooke S.T. , Witztum J.L., Bennet C.F., Baker B.F. RNA-targeted therapeutics. Cell Metab. 2020b; 27:714–739. 10.1016/j.cmet.2018.03.004 29617640

[35] Richard C. , Verdier F. Transferrin receptors in erythropoiesis. Int. J. Mol. Sci. 2020; 21:9713. 33352721 10.3390/ijms21249713 PMC7766611

