# Supplementary material for: Targeted tissue delivery of RNA therapeutics using antibody–oligonucleotide conjugates (AOCs)
Source: Nucleic Acids Res. 2023 May 24;51(12):5901–10. doi: 10.1093/nar/gkad415 (PMC10325888; doi:10.1093/nar/gkad415)
Supplement: gkad415_Supplemental_File [file gkad415_supplemental_file.docx]

**Targeted Tissue Delivery of RNA Therapeutics Using
Antibody-Oligonucleotide Conjugates (AOCs)**

**SUPPLEMENTAL TABLES AND FIGURE LEGENDS**

Supplemental Table 1: Amino Acid Sequence of αhTfR1.

Supplemental Table 2: Composition of Test Articles.

Supplemental Figure 1. Conjugation of siRNA Oligonucleotides to αASGR or αTfR1 Antibodies Had No Impact on Receptor Binding Affinity. An ELISA binding assay was developed using recombinant receptors (mouse TFRC/CD71 or ASGR1, Sino Biological) to determine the binding affinity (Kd) for the αASGR or αTfR1 antibodies toward ASGR or TfR1, respectively. Briefly, recombinant receptors were fixed onto plates (Costar 3690) followed by incubation with the free antibodies or AOCs (0.2–1,000 pM). A horseradish peroxidase (HRP) conjugated secondary antibody was utilized to detect binding based on chemiluminescence. Kd was calculated using the non-linear fit “Specific Binding with Hill Slope” method in GraphPad Prism software.

Supplemental Figure 2. Muscle Tissue Selectivity of αTfR1-si*SSB* Activity in the Cynomolgus Monkey. Male cynomolgus monkeys of Cambodian origin were administered a single IV dose of *SSB* siRNA conjugated to an αTfR1 at 6 mg/kg. Tissues were collected at 23 days post dose, and mRNA expression was analyzed by RT-qPCR. *SSB* expression was normalized to that of a reference gene, *AHSA1*. Data are represented as percent of vehicle control (mean; N=2).

Supplemental Figure 3. αASGR Conjugated to PMO Oligonucleotide Mediates *Pah* Exon 11 Skipping Activity in Liver. Male C57BL/6 mice were treated with a single IV dose of AOCs (5 mg/kg PMO), each composed of an antibody targeting mouse ASGR and conjugated to either a PMO designed for skipping of exon 11 of the Pah mRNA (αASGR-pmoPah, DAR 1.5) or a scrambled PMO (αASGR-pmoScr). The full length Pah mRNA has an expected size of 703 bp, and the skipped Pah Δ11 transcript is anticipated to be 569 bp. Data are represented as individual data points (N=2/group/timepoint). W: collection timepoint in weeks.

Supplemental Figure 4. AOC Demonstrates Improved Potency Relative to Unconjugated ASO Oligonucleotides in Muscle. Male C57BL/6 mice were administered an αTfR1 antibody conjugated to an ASO targeting the *Dmpk* mRNA (αTfR1-aso*Dmpk*) or the unconjugated ASO targeting *Dmpk* (aso*Dmpk*). A single dose of αTfR1-aso*Dmpk* was administered (0.6–9 mg/kg), while aso*Dmpk* was administered repeatedly for up to 2 weeks (cumulative dose of 9–300 mg/kg). Tissue samples were collected at
14 days post dose, and mRNA expression was analyzed by RT-qPCR. *Dmpk* mRNA expression was normalized to that of a reference gene, *Ppib*. Data are represented as percent of vehicle control (mean ± SEM; N=4 for treated groups, N=5 for vehicle group). Statistical analysis for target reduction was performed using one-way ANOVA and Dunnett’s *post hoc* test. Statistical difference relative to vehicle control at *p*<0.05 was observed for all treatments, except for 8.3 mg/kg aso*Dmpk*. Statistical analysis for the difference in potency was performed using ANCOVA test, demonstrating significantly lower ED_50_ value for AOC compared with unconjugated ASO with *p*<0.05.

Supplemental Figure 5. Plasma and Tissue Pharmacokinetics Profile of αTfR1-si*Mstn*, the Unconjugated si*Mstn* siRNA, and the Unconjugated αTfR1 Antibody. Male C57BL/6 mice were administered a single IV dose of αTfR1-si*Mstn*, si*Mstn*, or αTfR1 at 3 mg/kg.

A. Blood was collected at several timepoints post dose for siRNA or antibody concentrations determination using stem-loop qPCR or ligand binding ECL-based assays.

B. Tissue samples were collected at 96 hours post dose for measurement of siRNA concentrations using stem-loop qPCR-based assay. Data are represented as mean ± SD; N=4/group. Statistical analysis of si*Mstn* tissue concentrations was performed using multiple unpaired t-test. *Indicates statistical difference relative to siMstn group at *p*<0.05.

Supplemental Figure 6. hTfR1 Does Not Compete for Transferrin Binding and Lacks ADCC Activity.

A. An ELISA binding assay was developed using human transferrin receptor (Sino Biological) to determine the potential for αhTfR1 to interfere with transferrin binding. Briefly, recombinant human TfR1 was fixed onto plates (Costar 3690) followed by incubation with its endogenous transferrin ligand (0.5 nM) and unconjugated antibodies (0.1–1,000 nM). A horseradish peroxidase (HRP) conjugated secondary antibody was utilized to detect transferrin binding based on chemiluminescence. An antibody, AF2474 (R&D Systems), that binds to the same binding stie on TfR1 as transferrin, was tested as a positive control.

B. A cell-based ADCC assay was developed where TfR1 expressing HEL 92.1.7 cells were used as the target and human PBMCs as the effector cells. ADCC activity represents HEL 92.7 cell killing as measured by lactate dehydrogenase release. Data are shown as mean ± standard deviation (N=3–4).

**SUPPLEMENTAL DATA**

Supplemental Table 1. Amino Acid Sequence of αhTfR1.

| Heavy Chain | MGWSCIILFLVATATGVHSQVQLQQPGAELVKPGASVKLSCKASGYTFTNYWMHWVKQR PGQGLEWIGEINPINGRSNYGERFKTKATLTVDKSSSTAYMQLSSLTSEDSAVYYCARGTR AMHYWGQGTSVTVSSASTKGPSVFPLAPCSRSTSESTAALGCLVKDYFPEPVTVSWNSG ALTSGVHTFPAVLQSSGLYSLSSVVTVPSSNFGTQTYTCNVDHKPSNTKVDKTVERKCCV ECPPCPAPPVAGPSVFLFPPKPKDTLMISRTPEVTCVVVDVSHEDPEVQFNWYVDGVEVH NAKTKPREEQFNSTFRVVSVLTVVHQDWLNGKEYKCKVSNKGLPAPIEKTISKTKGQPREP QVYTLPPSREEMTKNQVSLTCLVKGFYPSDISVEWESNGQPENNYKTTPPMLDSDGSFFLY SKLTVDKSRWQQGNVFSCSVMHEALHNHYTQKSLSLSPGK |
| --- | --- |
| Light Chain | MGWSCIILFLVATATGVHSDIQMTQSPASLSVSVGETVTITCRTSENIYNNLAWYQQKQGKSP QLLVYAATNLADGVPSRFSGSGSGTQYSLKINSLQSEDFGNYYCQHFWGTPLTFGAGTKLEL KRTVAAPSVFIFPPSDEQLKSGTASVVCLLNNFYPREAKVQWKVDNALQSGNSQESVTEQDS KDSTYSLSSTLTLSKADYEKHKVYACEVTHQGLSSPVTKSFNRGEC |

Supplemental Table 2. Composition of Test Articles.

| **Test Article** | **Receptor Tissue Target** | **Tissue Gene Target** | **Sub-class** | **Sequence** |
| --- | --- | --- | --- | --- |
| **αASGR-si*Ctnnb1*** | ASGR - Liver | *Ctnnb1* | siRNA | (5'vp)UmsUfsUfsCoGfAoAfUfCoAfAoUfCoCfAoAfCoAoGosUosUo / (5'NH2C6)(5'iB)sCosUoGoUoUfGoGfAoUfUoGfAoUfUoCfGoAfAoAoUosUos(3'iB)(3'C6SSC6dT) |
| **αASGR-si*FVII*** |  | *FVII* |  | (5'vp)UmsUfsAfsAoGfAoCfUfUoGfAoGfAoUfGoAfUoCoCosUosUo / (5'NH2C6)(5'iB)sGosGoAoUoCfAoUfCoUfCoAfAoGfUoCfUoUfAoAoUosUos(3'iB)(3'C6SSC6dT) |
| **αASGR-si*Hprt*** |  | *Hprt* |  | (5'vp)UmsUfsAfsAoAfAoUfCfUoAfCoAfGoUfCoAfUoAoGosUosUo / (5'NH2C6)(5'iB)sCosUoAoUoGfAoCfUoGfUoAfGoAfUoUfUoUfAoAoUosUos(3'iB)(3'C6SSC6dT) |
| **αASGR-siScr** |  | NA^1^ |  | UosAosUfsCoGfAoCfGfUoGfUoCfCoAoGoCfUoAoGosUosUo / (5'NH2C6)(5'iB)sCosUoAoGoCfUoGfGoAfCoAfCoGfUoCfGoAfUoAoUosUos(3'iB)(3'C6SSC6dT) |
| **GalNAcASGR-si*FVII*** |  | *FVII* |  | (5'vp)UmsUfsAfsAoGfAoCfUfUoGfAoGfAoUfGoAfUoCoCosUosUo / (5’TriGalNAc)(5'iB)sGosGoAoUoCfAoUfCoUfCoAfAoGfUoCfUoUfAoAoUosUos(3'iB) |
| **αIgG1-si*Hprt*** | NA^2^ | *Hprt* |  | (5'vp)UmsUfsAfsAoAfAoUfCfUoAfCoAfGoUfCoAfUoAoGosUosUo / (5'NH2C6)(5'iB)sCosUoAoUoGfAoCfUoGfUoAfGoAfUoUfUoUfAoAoUosUos(3'iB)(3'C6SSC6dT) |
| **αTfR1-si*Ctnnb1*** | TfR1 - Muscle | *Ctnnb1* |  | (5'vp)UmsUfsUfsCoGfAoAfUfCoAfAoUfCoCfAoAfCoAoGosUosUo / (5'NH2C6)(5'iB)sCosUoGoUoUfGoGfAoUfUoGfAoUfUoCfGoAfAoAoUosUos(3'iB)(3'C6SSC6dT) |
| **αTfR1-si*Mstn*** |  | *Mstn* |  | (5'vp)UmsUfsAfsUoUfAoUfUfUoGfUoUfCoUfUoUfGoCoCosUosUo / (5'NH2C6)(5'iB)sGosGoCoAoAfAoGfAoAfCoAfAoAfUoAfAoUfAoAoUosUos(3'iB) |
| **αTfR1-siSsb** |  | *Ssb* |  | (5'vp)UqsUfsAoCoAoUfUoAoAoAoGoUoCoUfGoUfUoGoUosUosUo / (5'NH2C6)AosCosAoAoCoAoGfAfCfUoUoUoAoAoUoGoUosAosAo |
| **αhTfR1-si*Ssb*** |  | *Ssb* |  | (5'vp)UmsUfsAoCoAoUfUoAoAoAoGoUoCoUfGoUfUoGoUosUosUo / (5'NH2C6)AosCosAoAoCoAoGfAfCfUoUoUoAoAoUoGoUosAosAo |
| **αTfR1-si*Dmpk (fig3)*** |  | *Dmpk* |  | (5'vp)UmsAfsGoAoCoAfAoUoAoAoAoUoAoCfCoGfAoGoGosUosUo / (5'NH2C6)CosCosUoCoGoGoUfAfUfUoUoAoUoUoGoUoCosUosAo |
| **αTfR1-si*Dmpk (fig5)*** |  | *Dmpk* |  | (5'vp)UqsAfsAoUoAoCfCoGoAoGoGoAoAoUfGoUfCoGoGosUosUo / (5'NH2C6)CosCosGoAoCoAoUfUfCfCoUoCoGoGoUoAoUosUosAo |
| **αTfR1-siScr** |  | NA^1^ |  | UosAosUfsCoGfAoCfGfUoGfUoCfCoAoGoCfUoAoGosUosUo / (5'NH2C6)(5'iB)sCosUoAoGoCfUoGfGoAfCoAfCoGfUoCfGoAfUoAoUosUos(3'iB) |
| **αTfR1-aso*Dmpk*** |  | *Dmpk* | ASO | (5'NH2C6)AbsCbsAbsdAsdTsdAsdAsdAsdTsdAs[5MdC]s[5MdC]sdGsAbsGbsGb |
| **aso*Dmpk*** | NA | *Dmpk* | ASO | AbsCbsAbsdAsdTsdAsdAsdAsdTsdAs[5MdC]s[5MdC]sdGsAbsGbsGb |
| **Chol-si*Mstn*** | NA^3^ | *Mstn* | siRNA | (5'vp)UmsUfsAfsUoUfAoUfUfUoGfUoUfCoUfUoUfGoCoCosUosUo / (5'Chol)(5'iB)sGosGoCoAoAfAoGfAoAfCoAfAoAfUoAfAoUfAoAoUosUos(3'iB) |
| **si*Mstn*** | NA | *Mstn* | siRNA | (5'vp)UmsUfsAoUoUoAfUoUoUoGoUoUoCoUfUoUfGoCoCosUosUo / (5'NH2C6)GosGosCoAoAoAoGfAfAfCoAoAoAoUoAoAoUosAosAo |
| **αASGR-pmo*Pah*** | ASGR - Liver | *Pah* Exon11 | PMO | (5'NH2)ApTpCpCpTpCpTpTpTpGpGpTpApApCpCpTpCpApCpCpTpCpApCp(3'Acetyl) |
| **αASGR-pmoScr** | ASGR - Liver | NA^1^ | PMO | (5'NH2)CpGpGpTpGpTpGpTpGpTpApTpCpApTpTpCpTpCpTpApGpTpGpTp(3'Acetyl) |
| **αTfR1** | TfR1-Muscle | NA | NA | NA |
| ^1^Scrambled sequence with no known gene target  ^2^Isotype control antibody with no known receptor target  ^3^Conjugated to cholesterol  NA: not applicable  Xo=2’O-methyl, Xf=2’Fluoro, Xb=LNA, 5MdC=5-Methyl deoxycytidine, dX=DNA, iB=inverted abasic, vp=vinylphosphonate, m=2’methoxy-ethyl, q=2’methoxy-ethyl unlocked, s=phosphorothioate, Xp=morpholino phosphorodiamidate. | | | | |

Supplemental Figure 1. Conjugation of siRNA Oligonucleotides to αASGR or αTfR1 Antibodies Has No Impact on Receptor Binding Affinity.

Supplemental Figure 2. Muscle Tissue Selectivity of αTfR1-si*SSB* Activity in the Cynomolgus Monkey.

Supplemental Figure 3. αASGR Conjugated to PMO Oligonucleotide Mediates *Pah* Exon 11 Skipping Activity in Liver.


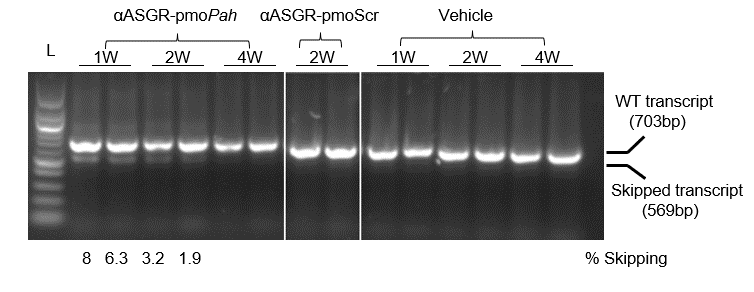


Supplemental Figure 4. AOC Demonstrates Improved Potency Relative to Unconjugated ASO Oligonucleotides in Muscle.

Supplemental Figure 5. Plasma and Tissue Pharmacokinetics Profile of αTfR1-si*Mstn,* the Unconjugated si*Mstn* siRNA, and the Unconjugated αTfR1 Antibody.

Supplemental Figure 6. hTfR1 Does Not Compete for Transferrin Binding and Lacks ADCC Activity.
